# Impact of Fibromyalgia on Alpha-2 EEG Power Spectrum in the Resting Condition: A Descriptive Correlational Study

**DOI:** 10.1155/2019/7851047

**Published:** 2019-04-01

**Authors:** Santos Villafaina, Daniel Collado-Mateo, Juan P. Fuentes-García, Ricardo Cano-Plasencia, Narcís Gusi

**Affiliations:** ^1^Faculty of Sport Science, University of Extremadura, Cáceres, Spain; ^2^Facultad de Educación, Universidad Autónoma de Chile, Talca, Chile; ^3^Clinical Neurophysiology, San Pedro de Alcántara Hospital, Cáceres, Spain

## Abstract

**Objective:**

The objective of this prospective cross-sectional study was to analyze the differences between patients with fibromyalgia and non-pain controls in terms of EEG power in the eyes-closed resting state. This study also aims to evaluate potential correlations between EEG power and subjective pain.

**Methods:**

The fibromyalgia patients were recruited by the Extremadura Association of Fibromyalgia (AFIBROEX) in Cáceres, Spain. Age- and sex-matched healthy controls (1:1 ratio) were recruited from university facilities and people close to the AFIBROEX by public calls. All underwent EEG during a 1-minute resting period with their eyes closed. The theta, alpha-1, alpha-2, beta-1, beta-2, and beta-3 frequency bands were analyzed by using EEGLAB. Self-reported visual analog scale pain scores were determined just prior to EEG.

**Results:**

A total of 62 women participated in the study, 31 of them diagnosed with fibromyalgia and 31 healthy controls. Fibromyalgia group exhibited a significantly lower alpha-2 in C4, T3, P4, Pz, and O2 compared to the healthy controls. Interestingly, pain correlated negatively with alpha-2 in Cz, P4, and Pz only in the fibromyalgia group.

**Conclusion:**

The fibromyalgia group exhibited decrease alpha-2 power in central, temporoparietal, and occipital brain areas. Furthermore, higher values of pain correlated with lower level of alpha-2 power in Cz, P4, and Pz. These findings may point the importance of alpha-2 power in pain in women with fibromyalgia.

## 1. Introduction

Fibromyalgia is a chronic syndrome that is characterized by widespread pain. Fatigue, stiffness, sleep disturbance, and affective and cognitive problems [1] are the most frequent symptoms. These aspects reduce the ability of fibromyalgia patients to perform daily life activities [2], thereby diminishing their quality of life [3].

Electroencephalography (EEG) power provides information about brain activity dynamics with excellent temporal resolution [4, 5]. Several neuroimaging and EEG studies propose that central nervous system hyperexcitability is an important mechanism in the maintenance of the chronic pain of fibromyalgia patients [6, 7]. Indeed, fibromyalgia patients differ from healthy controls in terms of their brain dynamics: in particular, fibromyalgia patients exhibit abnormal activation in the thalamic nuclei, somatosensory cortex, anterior cingulate, insula, and prefrontal cortices during pain processing [8–10]. Moreover, compared to healthy groups, fibromyalgia patients even display altered brain dynamics [11] even at rest [5, 12]. Fibromyalgia patients exhibit a greater power density in the beta band over the right middle frontal lobe and midcingulate gyrus [5] as well as significantly reduced delta, theta, and alpha bands in the frontal areas [12]. Navarro-López* et al.* (2015) proposed that the lower alpha EEG power band of fibromyalgia patients compared to healthy people during the closed-eye state may associate with diminished sensorimotor integration in brain processing and could, therefore, be an indicator of the need of fibromyalgia patients to make extra efforts to attenuate the chronic pain sensation [13].

This prospective cross-sectional study aims to contribute further to our understanding of the EEG power differences between fibromyalgia patients and healthy controls. Thus, EEG power spectrum analyses of FM patients and healthy controls in the eyes-closed resting state were conducted. Furthermore, this study also aims to investigate potential correlations between pain and EEG power spectrum. It was hypothesized that, in line with the studies discussed above, fibromyalgia patients would exhibit altered EEG patterns in this condition.

## 2. Materials and Methods

### 2.1. Selection of Participants

The Extremadura Association of Fibromyalgia (AFIBROEX) in Cáceres recruited the participants with fibromyalgia by telephone calls. All had been diagnosed with FM according to American College of Rheumatology criteria [1]. Participants with FM were included if they were on stable medication. Participants with fibromyalgia who had neurological diseases, psychiatric diagnosis (*i.e.*, major depression with suicidal ideation, schizophrenia, or substance abuse), and/or autoimmune or inflammatory diseases that can cause pain were excluded. In addition, healthy controls, who did not suffer from both localized or generalized chronic pain in the last six months, who were matched in terms of sex and age to the fibromyalgia participants (1:1 ratio) were also selected.

All participants were verbally informed about the details of the study and gave written informed consent to participate. All participants underwent EEG in July, 2017. All procedures were approved by the research ethics committee of the University of Extremadura (approval number: 62/1017) and were conducted in accordance with the tenets of the updated Declaration of Helsinki.

### 2.2. Procedures

All participants underwent a semi-standardized interview that assessed their medication intake. The pain intensity was also measured by using the visual analog scale (VAS) [14]. This is a unidimensional measure of pain intensity that consists in a straight line that is graded from 0 to 100, where 0 corresponds to “no pain at all” and 100 to “pain as bad as it could be”. All participants were asked to mark their pain level in the last week.

### 2.3. EEG Recording and Data Processing

The participants were instructed to rest on a chair in a quiet room with their eyes closed. The EEG signals were assessed during a 1-minute period by using the Enobio device, which is a wireless electrode system (Neuroelectrics, Cambridge, MA, USA) [15]. The reliability of this instrument has been validated, even when dry electrodes are used [16]. EEG was recorded from 19 scalp locations according to the International 10–20 system, namely, from seven frontal locations (Fz, Fp1, Fp2, F3, F4, F7, and F8), three central locations (Cz, C3, and C4), four temporal locations (T3, T4, T5, and T6), three parietal locations (Pz, P3, and P4), and two occipital locations (O1 and O2). Electrodes placed on the mastoids served as the references and impedance was kept below 10 KΩ. EEG was recorded with a sampling rate of 500 Hz, a 50 Hz notch filter, and bandpass filtering (1–40Hz). The EEGlab toolbox (MatLab) was used for pre-processing and data analysis. Rough artifacts were removed from the EEG signals and eye movement artifacts were corrected by using independent component analysis (ICA) [17]. The data were banded into the theta (4–7 Hz), alpha-1 (8–10 Hz), alpha-2 (11–12 Hz), beta-1 (13–18 Hz), beta-2 (19–21 Hz), and beta-3 (22–30) frequency bands. Data was computed using the function pop_spectopo.m from EEGlab. It allows us to export the mean *μ*V2/Hz per channel and frequency band to an excel file, being easy to correlate the EEG data with the behavioral variables such as VAS pain.

### 2.4. Statistical Analyses

The SPSS statistical package (version 20.0; SPSS, Inc., Chicago, Ill.) was used to analyze the data. Parametric tests were conducted based on the results of Shapiro-Wilk and Kolmogorov-Smirnov normality tests.

Independent* t-*tests were conducted to explore differences between groups in the six different frequency bands at each of the 19 channels. In addition, correlation in the fibromyalgia group between VAS pain scores and the power in the alpha-2 frequency band at each of the 19 channels was determined by Pearson's correlation coefficient analyses.

Furthermore, in order to examine the possible influence of age or use of medication (antidepressants and/or analgesics/relaxants) in the frequency bands, at each of the 19 channels, Pearson's correlation coefficient analyses and independent* t*-tests were respectively conducted.

The alpha-level of significance was set at 0.05.

## 3. Results

In total, 62 women participated in this cross-sectional study, namely, 31 women with fibromyalgia (mean ± standard deviation age = 54.52 ± 10.23 years) and 31 healthy controls (50.84 ± 8.51 years). Independent* t*-tests showed that the fibromyalgia and control groups differed significantly in terms of VAS pain scores (*p*<0.001) and frequency of antidepressant or analgesic/relaxant medication but did not differ significantly in terms of age (Table 1).

**Figure 1:**
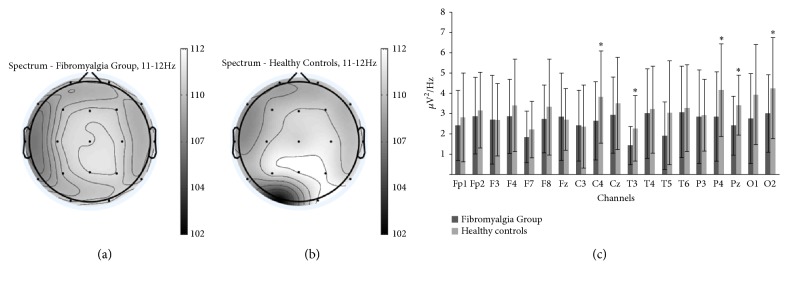
*Topographic maps of the power spectrum in the alpha-2 (11-12 Hz) frequency band for (a) fibromyalgia group (b), the healthy controls, and (c) the comparison of the fibromyalgia patients and the healthy controls in terms of alpha-2 power at 19 scalp channels*. The bars indicate standard deviation of the mean. ^*∗*^Significantly higher values (*p*<0.05) in the healthy controls* versus* the fibromyalgia group, as determined by independent* t*-tests.

Furthermore, independent* t*-tests comparing the fibromyalgia and the healthy controls, in terms of their EEG data, showed that the fibromyalgia patients had decreased alpha-2 power in C4 (*p*=0.049), T3 (*p*=0.018), P4 (*p*=0.043), Pz (*p*=0.028), and O2 (*p*=0.047) (see Figure 1). Other intergroup differences in EEG data were not found (data not shown).

Pearson's correlation coefficient analyses of the fibromyalgia group showed that their VAS pain scores correlated significantly and negatively with Alpha-2 at Cz, P4, and Pz (Table 2). Other significant correlations between pain level and EEG power were not found.

Interestingly, Pearson's correlation coefficient analyses of the 31 fibromyalgia women showed that age did not correlate with the theta, alpha-1, alpha-2, beta-1, beta-2, and beta-3 band signals at the 19 scalp locations. Moreover, independent* t*-tests also showed that the fibromyalgia patients who were treated with antidepressant and/or analgesic/relaxant medication did not differ from the fibromyalgia patients who were not being treated with these medications in terms of the theta, alpha-1, alpha-2, beta-1, beta-2, and beta-3 band signals at the 19 scalp locations.

## 4. Discussion

The present study examined the differences between fibromyalgia patients and non-pain controls in terms of their EEG power spectrum frequency bands (theta, alpha-1, alpha-2, beta-1, beta-2, and beta-3) at rest with their eyes closed. We found that the fibromyalgia patients had lower alpha-2 power in the central, temporoparietal, and occipital areas than the non-pain controls. Some of these results are in line with those reported in previous studies that focused on brain dynamics differences between fibromyalgia patients and non-pain controls. Specifically, these studies show that fibromyalgia patients have greater beta activity over the right middle frontal lobe and the midcingulate gyrus [5] and markedly lower delta, theta, and alpha activity in the frontal areas [12]. Significantly, however, the present study showed for the first time that, compared to non-pain controls, fibromyalgia patients also have significantly lower alpha-2 EEG power in posterior brain areas. This is consistent with the results observed by Hargrove* et al.* (2010) [12] for the entire alpha band.

Alpha synchronization or coherence is believed to be a communication mechanism in the brain. Specifically, alpha-2 oscillations associate with tonic alertness in a network that includes the dorsal anterior cingulate cortex, the anterior insula, and the anterior prefrontal cortex [18]. Vanneste* et al.* (2017) [19] suggest that functional connectivity between the posterior and dorsal anterior cingulate cortex in the alpha power band could indicate that a painful state has become part of the self-referential network [19]. This mechanism is known as allostatic reference resetting [20] and is hypothesized to underlie fibromyalgia [21]. Allostasis is defined as the adaptive response of the organism to maintain homeostasis to stressors. While allostasis has positive effects in the short term, too much stress and/or inefficient management of allostasis can lead to allostatic load or overload [22], namely, chronic increases in the levels of cortisol and catecholamines that cause structural remodeling of important brain structures such as the hippocampus [23], the amygdala [24], and the prefrontal cortex [25, 26]. These changes may relate to the learning and emotional deficits that are often observed in patients with chronic pain [27]. In this regard, several studies report that traumatic events related to chronic pain diseases like fibromyalgia [28–31]. These traumatic events may initiate the allostatic load and the resulting remodeling of the brain structures. This could explain the differences between the fibromyalgia patients and the healthy controls in terms of their brain dynamics.

Our findings support the use of therapies that could putatively remodel the brain dynamics of fibromyalgia patients. These therapies include neurofeedback, transcranial direct current stimulation (tDCS), and repetitive transcranial magnetic stimulation (rTMS). In relation to neurofeedback, however, a meta-analysis [32] suggests it has inconsistent outcomes in fibromyalgia patients [33–35]. By contrast, a recent meta-analysis showed that tDCS and rTMS may effectively reduce pain in fibromyalgia patients [36]. Moreover, a systematic review showed that anodal tDCS in the left primary motor cortex can be recommended as a treatment for fibromyalgia since it appears to modify the sensorial processing of the pain by the thalamic inhibitory circuitry [37]. Thus, by providing new information about the brain areas and the frequency bands that are altered in fibromyalgia, our results could help guide future neurofeedback, tDCS, and rTMS interventions.

Our study also showed that there was a negative correlation between the pain levels of the fibromyalgia patients and their alpha-2 power in the Cz, P4, and Pz EEG channels. In this regard, Nir* et al.* showed that, compared to the resting condition, healthy people placed in a noxious condition also exhibit decreases in the alpha-1 and alpha-2 bands. Moreover, like us, they showed that the parietal scalp channels correlated with pain levels [38]. This suggests that the chronic pain of fibromyalgia patients acts like a noxious stimulus and could modulate these channels over time, thereby eventually decreasing the alpha-2 power band in the resting condition. Given that only fibromyalgia patients exhibit decreased alpha-2 power in the resting condition [21], our results and those of Nir* et al.* [38] reinforce the hypothesis that allostatic reference resetting underlies fibromyalgia.

One potential limitation of our study design was the possible effect of pharmacological treatment on the results. However, independent* t*-tests comparing the fibromyalgia patients who were or were not taking medication (analgesics/relaxants and/or antidepressants) showed that the two subgroups did not differ significantly in terms of EEG results. Another limitation was that our sample was only composed of women. Thus, the results of this study cannot be generalized to male patients with fibromyalgia.

## 5. Conclusions

In summary, compared to non-pain control subjects, the fibromyalgia group exhibited a significant decrease in alpha-2 power in the central, temporoparietal, and occipital areas. The fibromyalgia group also exhibited negative correlations between their pain level and the alpha-2 frequency band in Cz, P4, and Pz. These findings indicate the importance of the alpha-2 frequency band in pain and the impact of fibromyalgia. Age did not relate significantly with the EEG data of the participants with fibromyalgia.

## Figures and Tables

**Table 1:** Demographics, pain, and medication use of the patients with fibromyalgia and the healthy controls just before electroencephalography.

Variable	Fibromyalgia group (Mean ± SD)	Healthy controls (Mean ± SD)
Sample size	31	31
Age	54.52 ± 10.23	50.84 ± 8.51
VAS of pain	59.03 ± 18.68	12.22 ± 17.83

Medication (No. of patients)		

Antidepressants	12	3
Analgesics/relaxants	10	0

SD, standard deviation; VAS, visual analog scale.

**Table 2:** Correlations between visual analog scale pain scores and alpha-2 power at the 19 electroencephalography scalp locations in the patients with fibromyalgia.

EEG channel	Pearson correlation coefficient	*p*-value^*∗*^
Fp1	-0.057	0.762
Fp2	0.123	0.509
F3	-0.081	0.665
F4	0.039	0.846
F7	-0.187	0.322
F8	-0.066	0.735
Fz	0.218	0.247
C3	-0.117	0.547
C4	-0.269	0.193
Cz	-0.463	0.015^*∗*^
T3	-0.228	0.225
T4	-0.008	0.968
T5	0.059	0.770
T6	-0.008	0.968
P3	0.029	0.884
P4	-0.422	0.040^*∗*^
Pz	-0.445	0.034^*∗*^
O1	-0.214	0.284
O2	-0.328	0.102

^*∗*^Significant (*p*<0.05), as determined by Pearson's correlation coefficient analysis

EEG, electroencephalography.

## Data Availability

The datasets generated during and/or analysed during the current study are available from the corresponding author on reasonable request.
